# Metabolic comparison of polycystic ovarian syndrome and control women in Middle Eastern and UK Caucasian populations

**DOI:** 10.1038/s41598-020-75109-2

**Published:** 2020-11-03

**Authors:** Alexandra E. Butler, Ahmed Abouseif, Soha R. Dargham, Thozhukat Sathyapalan, Stephen L. Atkin

**Affiliations:** 1 Diabetes Research Center (DRC), Qatar Biomedical Research Institute (QBRI), Hamad Bin Khalifa University (HBKU), Qatar Foundation (QF), PO Box 34110, Doha, Qatar; 2 Department of Obstetrics, Hamad Medical Corporation, Doha, Qatar; 3 Infectious Disease Epidemiology Group, Weill Cornell Medicine, PO Box 24144, Doha, Qatar; 4 Department of Academic Diabetes, Endocrinology and Metabolism, University of Hull, Hull, UK; 5 RCSI Bahrain, PO Box 15503, Busaiteen, Bahrain

**Keywords:** Endocrinology, Endocrine system and metabolic diseases

## Abstract

To determine if metabolic characteristics differed in women with and without polycystic ovary syndrome (PCOS) between a Caucasian and Middle East population. Comparative cross-sectional analysis. Demographic and metabolic data from Middle Eastern women from Qatar Biobank (97 with PCOS, 622 controls) were compared to a Caucasian PCOS biobank in Hull UK (108 with PCOS, 69 controls). In both populations, PCOS women showed a worse cardiovascular risk profile of increased systolic and diastolic blood pressure, increased C-reactive protein (CRP), reduced HDL, insulin resistance as well as increased androgens compared to their respective controls without PCOS. UK women without PCOS had higher systolic and diastolic blood pressures, and increased testosterone results (p < 0.01) compared to Middle Eastern women without PCOS who had higher inflammatory markers (WBC and CRP), HDL and insulin resistance (p < 0.001). UK PCOS women had a higher body mass index, systolic and diastolic blood pressures, triglycerides (p < 0.01), whilst Middle Eastern PCOS women showed increased testosterone, free androgen index, HDL and CRP (P < 0.01). There was no difference in insulin or insulin resistance between the two PCOS cohorts. This study highlights ethnic population differences because, whilst cardiovascular risk indices were increased for both PCOS cohorts, this may be for different reasons: BMI, waist and hip measurements, systolic and diastolic blood pressure, and triglycerides were higher in the UK cohort whilst testosterone, HDL and CRP were higher in the Middle East population. Insulin resistance did not differ between the two PCOS populations despite differences in BMI.

## Introduction

Polycystic ovary syndrome (PCOS) is the most common endocrine disorder that affects 6 (NIH criteria) − 10% (Rotterdam and Androgen excess society guidelines) of reproductive aged- women^1,2^ based on a Caucasian population. It has recently been shown that the prevalence based on the NIH criteria in Qatar in the Middle East may be higher than that of a Caucasian population^3^. PCOS leads to irregular periods, infertility and increased androgen levels causing hirsutism and acne^4,5^. Obesity affects the majority of women with PCOS, and they have a higher prevalence of both impaired glucose tolerance and type 2 diabetes^6,7^. Women with PCOS show increased cardiovascular risk through a higher incidence of hypertension, an adverse lipid profile, and insulin resistance (IR)^8,9^. Women with PCOS tend to have increased risk of cardiovascular disease, this risk seemingly increasing in relation to age^10^. It has been reported that women with PCOS have deficient insulin signaling which leads to insulin resistance and increased risk of type 2 diabetes (T2DM)^11^.

Differences in phenotype and clinical symptoms of PCOS related to the clinical, hormonal, and metabolic characteristics among various ethnic backgrounds, including Hispanics, African Americans, Asians, and Indians, have been described^12,13^. Differences in women of Middle Eastern origin have been described in comparison to Caucasian women^13^, with Caucasian women reported as having a more adverse cardiovascular profile with increased insulin sensitivity^14^, though the populations were not age matched; however, others have not found this to be so^15^.

From in silico analysis of the phenotype-genotype relationship, using single nucleotide polymorphisms determining the degree of genetic similarity, women from the Middle East are predicted to have a more severe hyperandrogenic phenotype^16^, but there are no comparative studies detailing the differences and similarities between a Caucasian and a homogeneous Middle Eastern population; therefore this study was undertaken.

## Methods

### Middle Eastern cohort

As detailed previously^3^ Qatar Biobank (QBB) https://www.qatarbiobank.org.qa is a large scale medical research initiative that aims to recruit 60,000 Qatari men and women aged ≥ 18 years. Following informed consent, extensive clinical phenotypic information was collected from each subject in addition to demographic, biochemical and genetic parameters that were approved by QBB IRB and the Ministry of Public Health in Qatar. 4500 subjects had been recruited aged between 18 and 89 years of age at the time of this study, of whom 719 Qatari women between the ages of 18–40 inclusive were included. Of those, 97 had PCOS and 622 did not. The diagnosis of PCOS for the comparison within the QBB was based on the NIH criteria of biochemical evidence of hyperandrogenemia (free androgen index > 4.5) or a raised testosterone greater than 2.7 nmol/l, and oligomenorrhea or amenorrhea. All identified PCOS subjects had no documented concurrent illness and were not on any medication; any illness or medication were exclusion criteria for the study.

### UK cohort

177 Caucasian women, 108 with PCOS and 69 without, who presented sequentially to the Department of Endocrinology, Hull and East Yorkshire Hospitals NHS Trust were recruited to the local PCOS biobank (ISRCTN70196169). All patients gave written informed consent. The Newcastle & North Tyneside Ethics committee approved this study. The diagnosis of PCOS was based on all three diagnostic criteria of the Rotterdam consensus, namely clinical or biochemical evidence of hyperandrogenism (Ferriman-Gallwey score > 8; free androgen index > 4.5 respectively), self-reported oligomenorrhea (≤ 9 menses per year) or amenorrhea (no menses for 3 months or more) and polycystic ovaries on transvaginal ultrasound (≥ 12 antral follicles in at least one ovary or ovarian volume of ≥ 10 cm^3^)^17^. Study participants had no concurrent illness, were not on any medication for the preceding 9 months and were not planning to conceive. For the purposes of this comparative study, all women fulfilled the NIH criteria for the diagnosis of PCOS.

In both cohorts, all women had prolactin, 17beta hydroxyprogesterone, thyroid function tests, and dehydroepiandrosterone sulfate (DHEAS) to exclude other confounding factors. Body mass index (BMI), waist circumference, height and weight were performed according to WHO guidelines. Control women had regular menses and normal androgen levels thus excluding PCOS by either NIH or Rotterdam criteria.

### Collection and analysis of blood samples

Blood samples were collected and were measured for the Middle Eastern population at the Chemistry Laboratory at Hamad Medical Corporation, Doha, Qatar, and for the UK population in the Chemistry Laboratory, Hull Royal Infirmary, UK that used the same laboratory platforms. TSH, prolactin, insulin, C reactive protein (CRP), DHEAS, and SHBG and measured by an immunometric assay with fluorescence detection on the DPC Immulite 2000 analyzer using the manufacturer’s recommended protocol, as previously described^1^. The Abbott testosterone method was performed for the Middle Eastern population within manufacturer’s specification throughout the sample collection (within run coefficient of variation (CV) 3.1%, within laboratory CV 3.6% at 2.6 nmol/L)^1^, whilst testosterone was measured by isotope dilution liquid chromatography-tandem mass spectrometry (Waters Corporation, Manchester, UK) in the UK, as previously described^9^.

“The free androgen index (FAI) was calculated as the total testosterone × 100/SHBG. Serum insulin was assayed using a competitive chemiluminescent immunoassay performed on the manufacturer’s DPC Immulite 2000 analyzer (Euro/DPC, Llanberis, UK). The analytical sensitivity of the insulin assay was 2 μU/ml, the coefficient of variation was 6%, and there was no stated cross-reactivity with proinsulin. Plasma glucose was measured using a Synchron LX 20 analyzer (Beckman-Coulter), using the manufacturer’s recommended protocol. The coefficient of variation for the assay was 1.2% at a mean glucose value of 5.3 mmol/L during the study period. The insulin resistance was calculated using the HOMA method [HOMA-IR = (insulin × glucose)/22.5]”^1^. All analyses were undertaken according to current guidelines, regulations and quality control.

### Statistical analysis

As described previously^4^, data trends were visually evaluated for each androgen and non-parametric tests were applied on data that violated the assumptions of normality when tested using the Kolmogorov–Smirnov Test. Accordingly, comparative analysis evaluating androgen levels between PCOS cases and controls was performed using the non-parametric Mann–Whitney test. Significance was defined at α = 0.05. All analyses were done using IBM-SPSS version 24.0. All values are given as (mean ± SD) unless specified. No formal power analysis was undertaken as there is not previous literature to do so.

### Ethics approval and consent to participate

The study was approved by the QBB IRB and the Ministry of Public Health in Qatar (Middle Eastern cohort) and the Newcastle & North Tyneside Ethics committee (UK cohort). All study participants signed an informed consent form prior to participation.

### Consent for publication

All authors gave their consent for publication.

## Results

The comparison between normal and PCOS subjects within the Middle East^3^ and UK^18^ subjects, respectively, within these databases for the increased cardiovascular risk indices (increased blood pressure, lower HDL, increased CRP) and increased androgens and insulin resistance seen in PCOS has been reported before.

When comparing the control women without PCOS between the Middle Eastern and UK cohorts (Table 1) it was seen that the age and BMIs were well matched, but waist measurement was higher in the UK (p < 0.04) whilst hip size was greater in the Middle Eastern population (p < 0.009). Systolic and diastolic blood pressure were higher in the UK cohort (p < 0.001), whilst testosterone (p < 0.02), glucose (p < 0.001), WCC (p < 0.001), HDL (P < 0.001), CRP (p < 0.001), insulin and insulin resistance (p < 0.001) were higher in the Middle Eastern cohort without PCOS.

**Table 1:** Comparison between the normal Caucasian UK (n = 69) and Middle Eastern (n = 622) population for subject demographics, cardiovascular and metabolic indices.

	UK population				Middle Eastern population				p-value
	Mean	Std. Dev	Median	Range	Mean	Std. Dev	Median	Range	
Age (years)	27.6	6.3	27.0	22.0	28.1	5.8	28.0	22.0	0.49
Height (cm)	167.5	6.9	168.0	38.0	160.1	6.4	160.6	41.2	< 0.001
Weight (kg)	97.1	23.8	94.0	106.7	78.0	16.5	80.5	81.8	< 0.001
BMI (kg/m^2^)	34.5	7.9	33.6	44.6	30.4	5.9	30.5	25.9	< 0.001
Waist (cm)	101.1	16.5	101.0	77.0	87.7	12.8	88.0	60.0	< 0.001
Hip (cm)	118.2	16.4	117.0	78.0	110.6	11.4	111.5	53.0	0.001
Systolic BP (mmHg)	121.8	14.4	120.0	74.0	109.0	10.9	109.0	53.0	< 0.001
Diastolic BP (mmHg)	77.1	10.7	77.0	54.0	72.8	7.9	72.0	45.0	0.001
Testosterone(nmol/l)	1.5	0.8	1.3	4.6	1.8	0.8	1.6	5.9	< 0.001
FAI	6.1	5.4	4.4	40.5	6.6	2.9	5.9	15.4	0.001
TSH (mU/l)	2.2	1.2	1.9	6.1	2.0	1.6	1.6	11.8	0.09
Glucose (mmol/l)	4.9	1.1	4.7	10.3	5.0	1.0	4.8	6.6	0.06
White cell count	7.1	2.0	6.9	10.8	7.5	1.9	7.4	9.2	0.07
Cholesterol (mmol/l)	4.8	1.0	4.6	4.4	4.7	0.7	4.6	3.8	0.72
Triglycerides(mmol/l)	1.6	1.3	1.2	8.4	1.2	0.6	1.0	2.6	0.02
HDL (mmol/l)	1.2	0.3	1.2	1.3	1.3	0.3	1.3	1.5	0.002
LDL (mmol/l)	2.9	0.9	2.8	4.1	2.8	0.7	2.9	3.7	0.91
CRP (mmol/l)	4.9	5.0	3.2	29.8	7.6	6.6	5.0	36.0	< 0.001
Insulin (μU/ml)	16.7	14.3	13.4	85.6	16.9	14.7	13.0	110.0	0.76
HOMA IR	4.1	6.0	2.7	54.9	4.0	4.3	2.6	32.1	0.54
SHBG (mmol/l)	33.4	21.2	27.0	110.6	28.5	10.7	27.0	58.0	0.67

*HDL* high density lipoprotein, *LDL* low density lipoprotein, *CRP* C reactive protein, *SHBG* sex hormone binding globulin.

When comparing the PCOS subjects (Table 2) it was seen that the BMI, waist and hip measurements, systolic and diastolic blood pressure (p < 0.001), and triglycerides (p < 0.015) were higher in the UK cohort. Testosterone (p < 0.001), HDL (P < 0.002), CRP (p < 0.001) were higher in the Middle Eastern population, whilst glucose, WCC, insulin and insulin resistance no longer differed between the 2 cohort of PCOS women.

**Table 2:** Comparison between the PCOS Caucasian UK (n = 108) and Middle Eastern (n = 97) population for subject demographics, cardiovascular and metabolic indices.

	UK population				Middle Eastern population				P-value
	Mean	Std. Dev	Median	Range	Mean	Std. Dev	Median	Range	
Age (years)	29.8	6.4	30.0	22.0	29.5	6.1	29.0	22.0	0.76
Height (cm)	166.2	5.8	166.0	29.0	159.2	6.0	159.4	34.8	< 0.001
Weight (kg)	73.1	14.1	70.1	57.7	67.9	16.7	65.0	107.6	0.002
BMI (kg/m^2^)	26.5	5.1	25.1	20.8	26.8	6.3	25.8	40.7	0.83
Waist (cm)	81.6	12.8	78.0	56.0	78.0	12.3	76.0	82.0	0.04
Hip (cm)	101.0	11.3	100.0	51.0	105.0	12.0	104.0	84.0	0.009
Systolic BP (mmHg)	114.7	11.8	113.0	57.0	104.6	10.9	104.0	76.0	< 0.001
Diastolic BP (mmHg)	73.3	11.5	74.0	57.0	68.7	9.4	68.0	79.0	< 0.001
Testosterone(nmol/l)	1.0	0.5	1.0	2.5	1.1	0.5	1.1	4.7	0.02
FAI	2.4	1.6	2.1	10.1	1.7	0.9	1.5	3.9	< 0.001
TSH (mU/l)	1.8	1.0	1.5	6.1	2.3	7.1	1.4	10.0	0.17
Glucose (mmol/l)	4.6	0.6	4.5	4.9	5.0	1.4	4.8	22.7	< 0.001
White cell count	5.7	1.4	5.5	5.6	6.7	1.8	6.5	12.1	< 0.001
Cholesterol (mmol/l)	4.6	0.7	4.7	3.3	4.8	0.8	4.7	5.4	0.35
Triglycerides(mmol/l)	1.1	0.8	0.8	4.6	1.0	0.5	0.8	3.7	0.57
HDL (mmol/l)	1.4	0.3	1.4	1.5	1.6	0.4	1.5	2.0	0.001
LDL (mmol/l)	2.7	0.6	2.6	2.9	2.8	0.7	2.8	5.0	0.89
CRP (mmol/l)	2.2	3.8	1.0	20.8	5.4	6.1	5.0	51.0	< 0.001
Insulin (μU/ml)	7.3	5.6	6.1	33.1	14.1	31.8	8.0	59.9	< 0.001
HOMA IR	1.6	1.5	1.2	9.2	3.6	12.4	1.7	26.4	< 0.001
SHBG (mmol/l)	50.0	22.6	45.0	104.0	85.0	61.5	67.0	46.1	< 0.001

*HDL* high density lipoprotein, *LDL* low density lipoprotein, *CRP* C reactive protein, *SHBG* sex hormone binding globulin.

## Discussion

These data comparing the Caucasian UK cohort with the homogeneous Middle Eastern cohort confirm the prediction that women of the Middle East have a more severe hyperandrogenic phenotype^16^ with an increased testosterone and FAI in women without PCOS and that is exacerbated in PCOS, highlighting the ethnic differences. These differences may in part contribute to the increase in hirsutism in the Middle East compared to Europe^13,19^.

The increased cardiovascular risk indices, increased androgen levels and increased insulin that result from PCOS compared to normal women has been reported for both the UK and Qatari databases^3,7,18^, indicating that there is an incremental worsening in these parameters irrespective of the ethnic baseline values, with PCOS. However, there were marked differences seen between the normal Caucasian and Middle Eastern women reported here. The Caucasian normal population had significantly higher systolic and diastolic blood pressures and a lower HDL that would suggest a comparatively higher cardiovascular risk and that would be in accord with others^14^. However, the Middle Eastern women without PCOS had increased markers of inflammation with a higher white cell count and higher CRP that has been associated with increased cardiovascular risk^20,21^. In addition, the Middle Eastern normal population had increased insulin levels and HOMA IR compared to the UK population, suggesting that they had an inherent increased insulin resistance despite their BMI and age being well matched.

In the comparison between the PCOS Caucasian and Middle Eastern populations, who were well matched for age, the differential in the insulin levels and HOMA seen in the normal population was lost and, in PCOS, both were increased, but did not differ between the populations. There was an increase in BMI for the Caucasian population that may have reflected the increase in systolic and diastolic blood pressure and triglycerides seen; however, the Middle Eastern population maintained the higher androgen levels for both testosterone and the FAI, and the CRP was significantly increased as seen in the normal population. This suggests that the Caucasian population may overall have higher cardiovascular risk^14^ compared to the Middle Eastern population. What this does suggest is that ethnically appropriate guidelines are needed for identifying anthropometric thresholds for better screening and diagnosis.

Glucose levels were significantly higher in the Middle Eastern normal compared to the Caucasian population. This differential was lost in PCOS where glucose levels did not differ between the populations, though were higher than for normal in accord with the increase in diabetes reported for PCOS^7^.

The strengths of this study were the relatively large cohorts for comparison that were age matched (and BMI matched in women without PCOS) and the complementary laboratory platforms for biochemical analysis; the exception to this was testosterone that was measured by immunoassay rather than gold standard tandem mass spectroscopy in the Middle East subjects that can be a problem due DHEAS cross reactivity in the immunoassay leading to potential assay interference y^22^, but the number of subjects would have mitigated against the relative imprecision of the assay. Both populations were homogeneous, being either Caucasian or Middle Eastern. The limitations of this study were that the UK population, by attending a secondary care facility, may have introduced an element of bias compared to the general population that was captured in the Qatar biobank. The inability to undertake a baseline power analysis decreases the evaluation of type 1 and type 2 statistical errors, but the relatively large numbers of subjects and also the very highly significant values found (p < 0.001) would make the chance of such errors very small.

In conclusion, this study highlights that, in these differing ethnic populations, that whilst cardiovascular risk indices were increased for both PCOS cohorts, this may be for different reasons: with BMI, waist and hip measurements, systolic and diastolic blood pressure and triglycerides higher in the UK cohort while testosterone, HDL and CRP were higher in the Middle Eastern population. Insulin resistance did not differ between the 2 PCOS populations despite differences in BMI.

## Data Availability

All the data for this study will be made available upon reasonable request to the corresponding author.

## References

[1] Legro RS, Arslanian SA, Ehrmann DA, Hoeger KM, Murad MH, Pasquali R (2013). Diagnosis and treatment of polycystic ovary syndrome: an Endocrine Society clinical practice guideline. J. Clin. Endocrinol. Metab..

[2] Yildiz BO, Bozdag G, Yapici Z, Esinler I, Yarali H (2012). Prevalence, phenotype and cardiometabolic risk of polycystic ovary syndrome under different diagnostic criteria. Hum. Reprod..

[3] Dargham SR, Ahmed L, Kilpatrick ES, Atkin SL (2017). The prevalence and metabolic characteristics of polycystic ovary syndrome in the Qatari population. PLoS ONE.

[4] Norman RJ, Dewailly D, Legro RS, Hickey TE (2007). Polycystic ovary syndrome. Lancet.

[5] Ehrmann DA (2005). Polycystic ovary syndrome. N. Engl. J. Med..

[6] March WA, Moore VM, Willson KJ, Phillips DI, Norman RJ, Davies MJ (2010). The prevalence of polycystic ovary syndrome in a community sample assessed under contrasting diagnostic criteria. Hum Reprod..

[7] Dargham SR, Shewehy AE, Dakroury Y, Kilpatrick ES, Atkin SL (2018). Prediabetes and diabetes in a cohort of Qatari women screened for polycystic ovary syndrome. Sci. Rep..

[8] Sathyapalan T, Atkin S. Recent Advances in the Cardiovascular Aspects of Polycystic Ovary Syndrome. European journal of endocrinology / European Federation of Endocrine Societies. 2011. 10.1530/EJE-11-0755 22096112

[9] Moran L, Teede H (2009). Metabolic features of the reproductive phenotypes of polycystic ovary syndrome. Hum. Reprod. Update..

[10] Atkin SL (2013). Cardiovascular disease in polycystic ovary syndrome. Clin. Endocrinol..

[11] Diamanti-Kandarakis E, Dunaif A (2012). Insulin resistance and the polycystic ovary syndrome revisited: an update on mechanisms and implications. Endocr. Rev..

[12] Wang S, Alvero R (2013). Racial and ethnic differences in physiology and clinical symptoms of polycystic ovary syndrome. Semin. Reprod. Med..

[13] Zhao Y, Qiao J (2013). Ethnic differences in the phenotypic expression of polycystic ovary syndrome. Steroids.

[14] Glintborg D, Mumm H, Hougaard D, Ravn P, Andersen M (2010). Ethnic differences in Rotterdam criteria and metabolic risk factors in a multiethnic group of women with PCOS studied in Denmark. Clin. Endocrinol..

[15] Al-Fozan H, Al-Futaisi A, Morris D, Tulandi T (2005). Insulin responses to the oral glucose tolerance test in women of different ethnicity with polycystic ovary syndrome. J. Obstet. Gynaecol. Can..

[16] Casarini L, Brigante G (2014). The polycystic ovary syndrome evolutionary paradox: a genome-wide association studies-based, in silico, evolutionary explanation. J. Clin. Endocrinol. Metab..

[17] Revised 2003 consensus on diagnostic criteria and long-term health risks related to polycystic ovary syndrome. Fertil Steril. 2004;81(1):19–25. 10.1016/j.fertnstert.2003.10.004 14711538

[18] Sathyapalan T, Al-Qaissi A, Kilpatrick ES, Dargham SR, Adaway J, Keevil B (2017). Salivary testosterone measurement in women with and without polycystic ovary syndrome. Sci. Rep..

[19] Al-Ruhaily AD, Malabu UH, Sulimani RA (2008). Hirsutism in Saudi females of reproductive age: a hospital-based study. Ann. Saudi Med..

[20] Ridker PM (2003). Clinical application of C-reactive protein for cardiovascular disease detection and prevention. Circulation.

[21] Ridker PM, Hennekens CH, Buring JE, Rifai N (2000). C-reactive protein and other markers of inflammation in the prediction of cardiovascular disease in women. N. Engl. J. Med..

[22] Warner MH, Kane JW, Atkin SL, Kilpatrick ES (2006). Dehydroepiandrosterone sulphate interferes with the Abbott Architect direct immunoassay for testosterone. Ann. Clin. Biochem..

